# AirSeg: Learnable Interconnected Attention Framework for Robust Airway Segmentation

**DOI:** 10.1007/s10278-025-01545-z

**Published:** 2025-05-22

**Authors:** Chetana Krishnan, Shah Hussain, Denise Stanford, Venkata Sthanam, Sandeep Bodduluri, S. Vamsee Raju, Steven M. Rowe, Harrison Kim

**Affiliations:** 1 Department of Biomedical Engineering, The University of Alabama at Birmingham, Birmingham, AL 35294 USA; 2 Department of Pulmonary, Allergy, and Critical Care, The University of Alabama at Birmingham, Birmingham, AL 35294 USA; 3 Department of Electrical Engineering, The University of Alabama at Birmingham, Birmingham, AL 35294 USA; 4 Department of Radiology, The University of Alabama at Birmingham, VH G082, 1720 2nd Avenue South, Birmingham, AL 35294 USA

**Keywords:** Multi-variant, Learnable, Embedding, Transformers, Attention, Airways, Medical image segmentation

## Abstract

Accurate airway segmentation is vital for diagnosing and managing lung diseases, yet it remains challenging due to data imbalance and difficulty detecting small airway branches. This study proposes AirSeg, a learnable interconnected attention framework incorporating advanced attention mechanisms and a learnable embedding module, to enhance airway segmentation accuracy in computed tomography (CT) images. The proposed framework integrates multiple attention mechanisms, including image, positional, semantic, self-channel, and cross-spatial attention, to refine feature representations at various network and data levels. Additionally, a learnable variance-based embedding module dynamically adjusts input features, improving robustness against spatial inconsistencies and noise. This improves the model’s robustness to spatial inconsistencies and noise, leading to more reliable segmentation results, especially in clinically challenging regions. AirSeg can be integrated with any UNet-like network with flexibility. The framework was evaluated on two datasets (in vivo and in situ) using several UNet-based architectures, comparing performance with and without AirSeg integration. Training employed data augmentation, a hybrid loss function combining Dice Similarity Coefficient and Intersection over Union losses, and statistical analysis to assess accuracy improvements. Integrating AirSeg into segmentation models led to statistically significant improvements in accuracy. Specifically, accuracy increased by 16.18% (*p* = 0.0035) for in vivo datasets and by 10.32% (*p* = 0.0097) for in situ datasets. These enhancements enable more precise identification of airway structures, including small branches, critical for early diagnosis and treatment planning in pulmonary care. The proposed model achieved a weighted average accuracy improvement of 12.43% (*p* = 0.0004) over other conventional models. AirSeg demonstrated superior performance in capturing both global structures and fine details, effectively segmenting large airways and intricate branches. Ablation studies validated the contributions and impact of individual attention mechanisms and the embedding module. The improvement in accuracy translates to more precise airway segmentation, enhancing the detection of small branches crucial for early diagnosis and treatment planning. The statistically significant *p*-values confirm that these gains are reliable, reducing manual correction efforts and improving the efficiency of automated airway analysis in clinical settings.

## Introduction

Accurate airway segmentation is crucial for anatomical characterization and phenotyping of airway morphological changes associated with pulmonary disorders [1]. However, manual delineation of airways is labor intensive, prone to variability among clinicians, and often impractical in many clinical settings [2]. To overcome these limitations, researchers have developed automated techniques for airway segmentation using computed tomography (CT) images [3]. Despite significant advancements, detecting small airway branches less than 2 mm, such as terminal bronchioles, remains a persistent challenge due to their small size and limited visibility on CT images. This difficulty is primarily due to substantial variations in voxel densities and pronounced data imbalance between smaller and larger branches [4]. Consequently, machine learning models often generate fragmented or false-negative predictions, mainly when applied to diverse patient populations with varying pulmonary conditions [5]. These challenges highlight the critical need for long-range representative learning for accurate airway segmentation.

Airway segmentation is inherently challenging due to data imbalance, anatomical variability, and the high similarity between airway structures and surrounding tissues. A pronounced disparity exists between foreground and background samples as well as between larger airways and smaller terminal branches. This imbalance complicates the training of data-driven models, making the accurate detection of terminal branches particularly challenging [6]. Although many neural networks use patch-based training, the methodologies for extracting these patches remain underexplored [7]. For instance, van Rikxoort et al. employed sliding window approaches without considering alternative sampling strategies [8]. In addition, the high degree of similarity in voxel intensities between airway structures and diseased tissue presents another challenge, making distinguishing airways from surrounding regions difficult [9]. Mucus impaction of airways, which can approach the voxel density of surrounding airway tissue, is another complexity (and of biological interest). Addressing this issue necessitates models incorporating additional spatial and structural contexts beyond voxel intensity information.

Fully convolutional neural networks (FCNs) and encoder-decoder models (UNet) are widely utilized for segmentation problems, including medical imaging [10]. These models typically consist of two main components: an up-sampling path, which compresses input data into compact representations, and a down-sampling path, which reconstructs labels either in a single or multiple stages [11]. Although effective for representation learning, these architectures often exhibit inefficiencies in information utilization. For instance, low-level features may be redundantly extracted at multiple stages, and the learned feature representations may lack the discriminative power necessary for pixel-level recognition in complex tasks [12].

Recent advancements have aimed to enhance feature discrimination through multi-scale contextual fusion. For example, Wang et al. introduced a pyramid-based network that aggregates local information at various levels using multiple dilated convolutional blocks [13]. Other approaches leverage pooling operations to combine multi-scale contextual information [14–16]. Although these strategies improve the ability to capture objects across varying scales, contextual dependencies are often treated uniformly across image regions, neglecting the unique needs of local representations and category-specific dependencies [17]. Furthermore, the design of multi-context representations in these methods is largely manual, which limits their flexibility and adaptability. This manual design approach also hinders the ability to fully exploit long-range relationships across an image, which is crucial for accurate medical imaging segmentation [18].

Attention mechanisms have emerged as powerful tools alongside deep convolutional neural networks, offering efficient ways to integrate local and global features for various computer vision tasks [19]. Unlike traditional feature integration approaches, which condense an image into a fixed representation, attention mechanisms dynamically prioritize the most relevant features [20]. This process reduces the redundancy in feature maps and emphasizes task-specific features without requiring additional supervision. For instance, Yang et al. introduced an attention mechanism that assigns weights to multi-scale features extracted at different resolutions for electron segmentation from microscopic images [21]. This approach outperformed traditional average and max-pooling methods for merging multi-scale feature predictions [22, 23], demonstrating the potential of attention mechanisms to refine feature integration and enhance segmentation outcomes.

Further innovations, such as the squeeze-and-excitation (SE) block, explicitly model interdependencies across channels of convolutional feature maps [24]. By adaptively recalibrating channel-wise feature responses, the SE block enhances the representational power of the network. Building on this concept, the Convolutional Block Attention Module (CBAM) extended attention modeling to both channel and spatial dimensions, enabling a more comprehensive focus on salient features [25]. The Bottleneck Attention Module (BAM) further integrated channel and spatial attention into a unified 3D attention map, offering greater flexibility and precision [26]. Beyond these modules, self-attention, cross-attention, and their derivatives have gained prominence, particularly in Vision Transformers (ViT), allowing networks to capture long-range dependencies and complex feature interactions for diverse vision tasks [27].

Despite these advancements, single-stage attention networks often include noise from irrelevant regions, which can degrade performance [28]. These methods frequently lack mechanisms to fully filter unrelated information during feature extraction and struggle to effectively integrate low-level and high-level features while preserving feature consistency throughout the network [29]. As a result, the richness and discriminative power of the generated feature representations are constrained, limiting the accuracy of localizing target regions, especially in challenging tasks such as small-object segmentation and airways [30].

To overcome these limitations, we explore advanced attention mechanisms that enhance the learning capacity and performance of deep networks for medical image segmentation. Unlike conventional methods that rely on either a single attention mechanism or multi-scale strategies, we propose AirSeg, a hybrid attention module designed to work within a unified framework. By integrating multiple attention mechanisms, AirSeg progressively refines feature representations, suppressing irrelevant information while emphasizing critical structural details. The combination of self-attention and cross-attention mechanisms, along with a learning module, enables the network to effectively capture both local and global dependencies, leading to more precise and reliable airway segmentation across varying scales and resolutions. These help in overcoming the segmentation problem despite the data imbalance.

## Materials and Methods

### Proposed Architecture

**Fig. 1:**
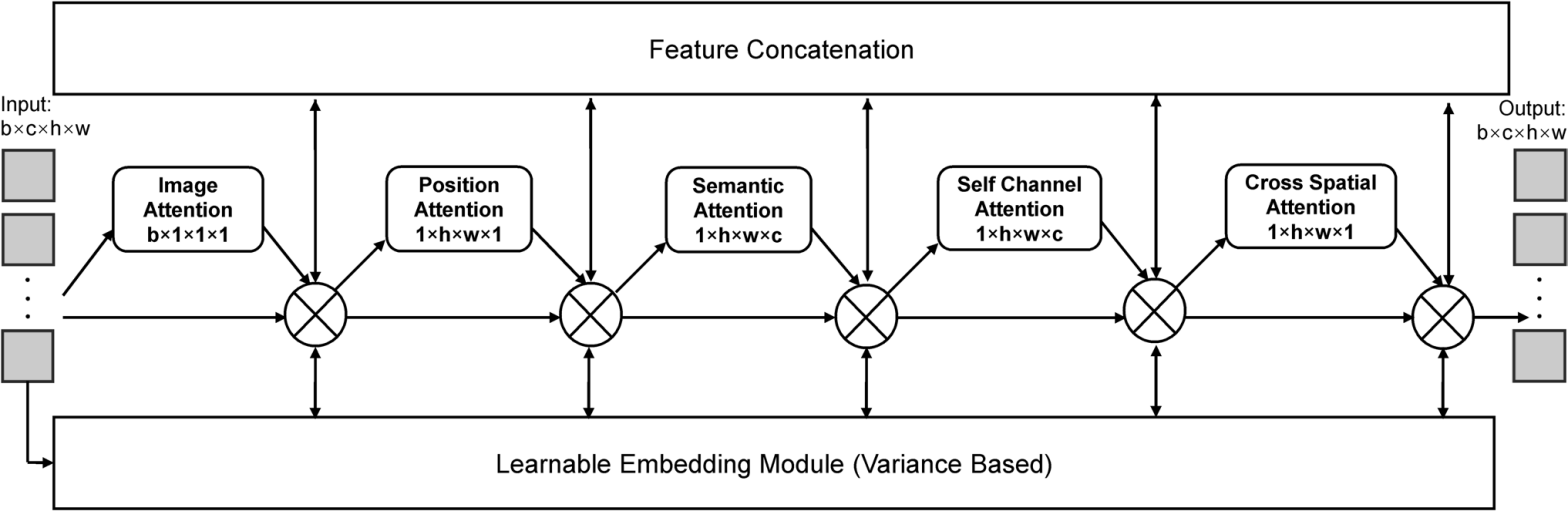
Schematic of the AirSeg Framework. The AirSeg module is designed to refine feature representations for enhanced 2D image segmentation by focusing on semantic, positional, and spatial channel-specific dependencies

Figure 1 shows the architecture of the proposed AirSeg Module, which was designed to refine feature representations for enhanced 2D image segmentation by focusing on semantic, positional, and spatial channel-specific dependencies. By processing feature maps at different levels of the network, AirSeg enhances the ability to highlight relevant regions while suppressing noise, leading to improved segmentation accuracy. Given input feature maps $F \in {R}^{h \times w \times c}$, where $h$ and $w$ denote the spatial dimensions and $c$ represents the number of channels, the module sequentially computes attention maps to emphasize important information. Image Attention $\left({M}_{\mathrm{image}}\in {R}^{1\times 1\times 1}\right)$ identifies the task-specific regions in the background. Positional attention $\left({M}_{\mathrm{positional}}\in {R}^{h\times w\times 1}\right)$ focuses on specific locations within each slice to emphasize the relevant spatial positions within individual slices. Semantic attention $\left({M}_{\mathrm{semantic}}\in {R}^{1\times 1\times c}\right)$ captures the global relevance of features across the entire input. This helps highlight the most significant features in the context of overall data. Additionally, self-channel attention $\left({M}_{\mathrm{schannel}}\in {R}^{1\times 1\times c}\right)$ refines feature maps by focusing on relationships within channels within each spatial location, allowing the model to understand channel interactions better. This differs from semantic attention, which is more concerned with the global importance of features regardless of their channel relationships. Finally, the cross-spatial attention $\left({M}_{\mathrm{cspatial}}\in {R}^{h\times w\times 1}\right)$, in contrast to position attention, captures interactions and dependencies across different spatial locations throughout the image, thus enhancing overall spatial reasoning. The refined feature maps, ${F}^{\prime}$, are calculated through sequential applications of these attention mechanisms as follows:

$${F}^{\prime}={{M}_{\text{image }}\otimes (M}_{\mathrm{positional}}\otimes \left({M}_{\mathrm{semantic}}\otimes ({M}_{\mathrm{schannel}}\otimes ({M}_{\mathrm{cspatial}}\otimes F)\right))$$

where $\otimes$ denotes element-wise multiplication and intermediate feature representations. Permutation was applied as required to align the dimensions during computation. On a parallel approach, the learnable embedding module processes the feature maps dynamically over each attention module, and hence, the final equation would be:

$${F}^{\prime}=\left({M}_{\mathrm{image}}\otimes {LEM}_{\mathrm{image}}\right)\otimes \left({M}_{\mathrm{positional}}\otimes {LEM}_{\mathrm{positional}}\right)\otimes \cdots \otimes \left({M}_{\mathrm{cspatial}}\otimes {LEM}_{\mathrm{cspatial}}\right)\otimes F$$

Where LEM corresponds to embeddings from each attention module. This ensures that LEM is applied at every stage of attention processing.

## Image Attention Module

**Fig. 2:**
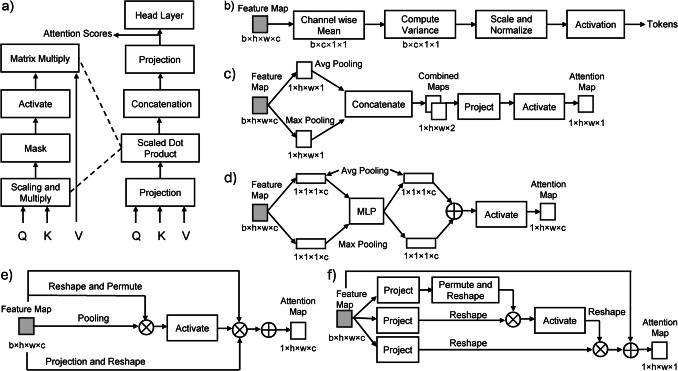
AirSeg sub-components. Schematics of (**a**) the image attention module (IAM), (**b**) the learnable variance-based embedding module (LEM), (**c**) the position attention module (PAM), (**d**) the semantic attention module (SAM), (**e**) the self-channel attention module (SCAM), and (**f**) the cross-spatial attention module (CSAM) parallel processed by the learning embedding module

Figure 2a illustrates the image attention module. The IAM module is engineered to pinpoint which pixels or groups will most likely hold essential information pertinent to the task while minimizing features that could result in false-positive predictions in specific regions. For instance, the module is expected to infer that positive airway segmentation is less probable in the corners of slices than in the center. The module also incorporates a regularization mechanism to address uncertainty within the input data, thereby improving robustness and generalization. This is achieved using a multi-head attention mechanism that allows the network to process diverse patterns simultaneously. IAM begins by dividing the input feature dimension across multiple attention heads. The input feature maps $x\in {R}^{B\times N\times C}$, where $B$ is the batch size, $N$ is the sequence length (flattened spatial dimensions), and $C$ is the feature dimension, are projected into three separate spaces: queries $\left(Q\right)$, keys $\left(K\right)$, and values $\left(V\right)$. This projection is achieved using a linear transformation: $Q,K,V={\mathrm{Linear}}\left(x\right)$, where $Q,K,V\in {R}^{B\times N\times C}$ represents distinct views of the input. The resulting tensors are reshaped and permuted for parallel processing across $H$, the number of attention heads, resulting in $Q,K,V=Q,K,V\in {R}^{B\times H\times N\times D}$, where $D = C / H$ is the dimension of each attention head. The attention scores are computed using the scaled dot product between the query (Q) and the transposed key, $\left({K}^{T}\right)$:$\mathrm{Scores}=\frac{{QK}^{T}}{\sqrt{D}}$. The scaling factor $\sqrt{D}$, ensures stable gradients during backpropagation. The scores are then normalized using a SoftMax function, which transforms them into a probability distribution over the keys for each query. Dropout is applied to these normalized attention scores to introduce regularization, preventing overfitting by randomly dropping some scores during training. Next, the weighted sum of the values, calculated using the attention scores, is computed, producing the attended output for each head. This output is transposed and reshaped back into the original input dimension. Finally, the output passes through another linear transformation and a dropout layer before being returned as the final output of the attention mechanism. This process allows the model to learn which parts of the input sequence are most relevant for the task, leveraging the parallelism of multi-head attention to capture diverse dependencies within the data.

### Learnable Embedding Module

Conventional embedding methods use convolutional layers or patch-wise techniques, leading to a loss of spatial coherence between adjacent patches and may not always effectively address normalization issues across different image regions [31, 32]. This can result in variations in feature scaling and activation, potentially impacting the uniformity of the features extracted. This is particularly problematic for tasks like image processing, where spatial and local features can vary significantly across samples. Second, traditional embeddings often ignore spatial relationships or local statistics, which are crucial in tasks like segmentation, where the context of a pixel or region significantly impacts its representation [33]. Lastly, these embeddings are susceptible to overfitting, as they rely heavily on static mappings without considering input-specific variations, making them less robust in diverse or noisy datasets [34]. Hence, we propose a learnable adaptive embedding as seen in Fig. 2b, designed to dynamically embed feature maps by leveraging spatial statistics such as mean and variance, as seen in Fig. 2b. This method begins with a 4D input tensor $x\in {R}^{B\times C\times H\times W}$, where $B$ is the batch size, $C$ is the number of channels, and $H,W$ are the spatial dimensions. To adaptively adjust the input features, the module first computes the mean of each feature channel across the spatial dimensions. This is expressed as $\upmu \left(x\right)=\frac{1}{H\cdot W}{\sum }_{i=1}^{H}{\sum }_{j=1}^{W}{x}_{:,:,i,j}$, resulting in a mean tensor of shape ${R}^{B\times C\times 1\times 1}$. The mean provides a baseline representation for each channel.

The variance of the input features is computed next, using the squared deviations from the mean. For each feature channel, the deviation is ${x}_{\mathrm{deviation}}={\left(x-\upmu \left(x\right)\right)}^{2}$. To stabilize the variance calculation, the deviations are summed and normalized by the number of elements minus one, giving ${\upsigma }^{2}\left(x\right)=\frac{1}{H\cdot W-1}{\sum }_{i=1}^{H}{\sum }_{j=1}^{W}{x}_{\mathrm{deviation}}+\upepsilon$, where $\epsilon$ is a small constant to prevent division by zero. This variance-like term captures the dispersion of feature values in the spatial dimensions, allowing the embedding to adapt dynamically to variations in the input. To create the embedding, a scaling factor $y$ is computed using the variance-like term and the feature deviations. The scaling formula is $y=\frac{{x}_{\mathrm{deviation}}}{4{\upsigma }^{2}\left(x\right)}+0.5$, where factor $4$ controls sensitivity to variance and $0.5$ ensures the scaling is centered around a neutral value. This step normalizes and stabilizes the feature adjustments. A sigmoid activation is then applied to $y$, resulting in ${y}^{\prime}={\mathrm{Sigmoid}}\left(y\right)$. The sigmoid function constrains the scaling factors to the range (0, 1), ensuring smooth and bounded adjustments.

The final embedding is computed by reweighting the original input tensor $x$ with the activated scaling factors $y^{\prime}$. This is expressed as ${x}_{\mathrm{embedded}}=x\cdot {y}^{\prime}$. The reweighting emphasizes spatial regions and channels that are more relevant to the task while suppressing less significant features. This dynamic adjustment enhances the feature representation, making it more robust to noise and spatial inconsistencies while passed on to the attention modules. In this manuscript,"embedding"refers to a learnable module that encodes local variance into feature space representations and is unrelated to token or position embeddings used in Transformer-based NLP models.

### Position Attention Module

The schematic of the Position Attention Module is illustrated in Fig. 2c. The Positional Attention Module processes the feature maps from the IAM, ${F}_{1}$, and generates a positional attention map ${M}_{\mathrm{positional}}$, which highlights key spatial regions. Feature maps are created for max pooling and average pooling operations along the channel dimension $(c)$. This results in two feature maps of the size of $1 \times h \times w$, each capturing complementary spatial information. The pooled feature maps are concatenated along the channel axis to create a combined representation. A convolution operation is then applied to this concatenated map to extract spatially relevant patterns. Finally, a sigmoid activation function is used to produce the positional attention map, ${M}_{\mathrm{positional}}$, expressed mathematically as ${M}_{\mathrm{positional}}={\mathrm{Sigmoid}}\left({\mathrm{Conv}}\left(\left[{\mathrm{MaxPool}}\left({F}_{1}\right);{\mathrm{AvgPool}}\left({F}_{1}\right)\right]\right)\right)$, where ${\mathrm{Conv}}\left[\cdot ;\cdot \right]$ denotes the convolution operation, and the semicolon indicates the concatenation of the max-pooled and average-pooled feature maps. The output from PAM is passed on to the next module.

### Semantic Attention Module

The Semantic Attention Module (Fig. 2d) processes the input feature maps $F$ from the PAM to generate a semantic attention map, ${M}_{\mathrm{semantic}}$, emphasizing the most important semantic information across all 2D feature maps in the input. Unlike typical 2D approaches, the semantic attention map remains consistent for a single channel across all spatial locations within a slice. This ensures a unified focus on semantic importance across the feature maps. To compute ${M}_{\mathrm{semantic}}$, global max pooling $\left({\mathrm{MP}}\left(\cdot \right)\right)$ and average pooling $\left({\mathrm{AP}}\left(\cdot \right)\right)$ are performed on the input feature maps across the spatial dimensions $\left(h,w\right)$. These pooling operations extract complementary information about the features, condensing the input $F\in {R}^{l\times h\times w\times c}$ into a reduced representation of ${R}^{1\times 1\times 1\times c}$. The resulting pooled features are then independently passed through a shared multi-layer perceptron (MLP) to learn attention weights. The outputs of the MLP for the max- and average-pooled features are summed, and a sigmoid activation function $\left(\upsigma \left(\cdot \right)\right)$ is applied to produce the semantic attention map, ${M}_{\mathrm{semantic}}=\upsigma \left({\mathrm{MLP}}\left({\mathrm{MP}}\left(F\right)\right)+{\mathrm{MLP}}\left({\mathrm{AP}}\left(F\right)\right)\right)$. This operation assigns a single attention weight per channel, ensuring that all spatial locations within a channel share the same semantic importance. By condensing spatial information into a channel-wise attention map, this module identifies which semantic features are most relevant to the task while ensuring consistency across slices or regions. This improves the model's ability to focus on semantically significant features and enhances its performance in tasks requiring high-level understanding. The calculated features are passed on to the next module.

### Self-channel Attention Module

The Self-channel Attention Module (SCAM; Fig. 2e) is designed to enhance feature representations by adaptively reweighting the importance of each channel in the input feature maps. Unlike SAM, which assigns consistent weights across spatial dimensions within each channel to emphasize overall semantic importance, SCAM focuses on capturing inter-channel relationships and dynamically adjusts the contribution of each channel based on its content. The input to the SCAM module is a feature map from SAM $x\in {R}^{B\times C\times H\times W}$, where $B$ is the batch size, $C$ is the number of channels, and $H,W$ are the spatial dimensions. To compute the self-channel attention, the module uses both global average pooling and global max pooling to extract complementary information about each channel. These pooling operations condense the spatial dimensions, producing two 1D vectors of shape, ${R}^{B\times C\times 1\times 1}$, which represents the overall content of each channel.

The SCAM module passes the condensed information through a two-layer, fully convolutional network for both the average-pooled and max-pooled representations. The first convolutional layer reduces the number of channels by a factor of ${\mathrm{ratio}}$ (a hyperparameter, typically set to 16), capturing inter-channel dependencies in a compressed space. This is followed by a ReLU activation to introduce non-linearity. The second convolutional layer restores the number of channels to the original $C$, allowing the module to model the relative importance of each channel in the context of the input data. The results of the two branches (average and max) are summed to combine complementary information, and a sigmoid activation is applied to produce the final self-channel attention map,

$${M}_{\mathrm{SCAM}}=\upsigma \left({\mathrm{Conv2D}}_{2}\left({\mathrm{ReLU}}\left({\mathrm{Conv2D}}_{1}\left({\mathrm{AvgPool}}\left(x\right)\right)\right)\right)+ {\mathrm{Conv2D}}_{2}\left({\mathrm{ReLU}}\left({\mathrm{Conv2D}}_{1}\left({\mathrm{MaxPool}}\left(x\right)\right)\right)\right)\right)$$

Where $\upsigma \left(\cdot \right)$ is the sigmoid function, and ${\mathrm{Conv2D}}_{1}$ and ${\mathrm{Conv2D}}_{2}$ represent the two convolutional layers. The resulting attention map ${M}_{\mathrm{SCAM}}\in {R}^{B\times C\times 1\times 1}$ contains a unique attention weight for each channel, dynamically reweighting the input feature maps by ${x}_{\mathrm{out}}=x\cdot {M}_{\mathrm{SCAM}}$ where $\cdot$ denotes element-wise multiplication, broadcasting the attention weights across the spatial dimensions. The output maps are passed on to the next module.

## Cross-Spatial Attention Module

The Cross-Spatial Attention Module (CSAM) (Fig. 2f) refines single-channel feature maps from SCAM by dynamically emphasizing spatial regions most relevant to the task. Given an input tensor $x\in {R}^{B\times 1\times H\times W}$, where $B$ is the batch size and $H,W$ are the spatial dimensions, the module computes a spatial attention map based on global pooling operations. The module first applies average and max pooling across the single channel dimension to capture complementary spatial information. The average pooling operation computes the mean value across all spatial locations for each pixel, given by ${x}_{\mathrm{avg}}\left(i,j\right)=\frac{1}{H\times W}{\sum }_{i=1}^{H}{\sum }_{j=1}^{W}{x}_{:,1,i,j}$. Similarly, max pooling determines the maximum value across all spatial locations, represented as ${x}_{\mathrm{max}}\left(i,j\right)=\underset{i,j}{\mathrm{max}}{x}_{:,1,i,j}$. These operations produce feature maps, ${x}_{\mathrm{avg}}$ and ${x}_{\mathrm{max}}$ of shape ${R}^{B\times 1\times H\times W}$. The two pooled outputs, ${x}_{\mathrm{avg}}$ and ${x}_{\mathrm{max}}$, are concatenated along the channel dimension to form a combined representation ${x}_{\mathrm{concat}}\in {R}^{B\times 2\times H\times W}$. This concatenation integrates complementary spatial information from both pooling operations, enabling the module to consider both average and peak activations when determining spatial importance. The concatenated feature map is then processed by a convolutional layer with kernel size $k$ (typically $k=3 \text{or }k=7$), which extracts spatial patterns and generates the attention map. The convolutional output is passed through a sigmoid activation function, ${M}_{\mathrm{CSAM}}=\upsigma \left({\mathrm{Conv2D}}\left({x}_{\mathrm{concat}}\right)\right)$, where $\sigma \left(\cdot \right)$ ensures that the attention values lie in the range $\left[\mathrm{0,1}\right]$. The resulting attention map ${M}_{\mathrm{CSAM}}\in {R}^{B\times 1\times H\times W}$ represents the relative importance of each spatial location. The spatial attention map is then applied element-wise to the original input feature map to refine it, amplifying important regions and suppressing less relevant ones. The refined feature map is computed as ${x}_{\mathrm{refined}}=x\cdot {M}_{\mathrm{CSAM}}$, where the dot denotes element-wise multiplication, and broadcasting ensures compatibility of dimensions. This final output ${x}_{\mathrm{refined}}\in {R}^{B\times 1\times H\times W}$ contains the reweighted spatial features, with task-relevant regions emphasized for improved downstream performance.

### Datasets

Non-contrast CT scans were conducted using a μCT scanner dedicated to small animal imaging (MiLabs, Utrecht, Netherlands). Ferrets were chosen because they are an intermediate-sized animal model and better recapitulate human lung disease due to the presence of terminal bronchioles and cell types that are representative of human diseases. All ferrets were anesthetized with inhaled isoflurane and prospectively gated for a single inspiratory phase of respiration. All images were acquired at ultra-focused magnification with respiratory gating using 55 kV of tube voltage, 0.19 mA of tube current, 1° of angular resolution, and 20 ms of exposure. All images were reconstructed using vendor software at 80-μm voxel resolution. Post-reconstruction, images were filtered using a 0.5-mm Gaussian smoothing kernel on OD software (PMOD Technologies LLC, Zurich, Switzerland). The ground truth airway was determined manually using the threshold and region-growing methods. Each 3D CT volume contained more than 500 slices. The images were sliced for 2D training. Out of 44 in situ 3D images (*n*=51,124 2D image slices), 34 were used for training (*n*=39,920), and the remaining 10 images were used for testing (*n*=11,204). Similarly, out of 71 in vivo 3D images (*n*=69,905 slices), 59 were used for training (*n*=57,683), and the remaining 12 were used for testing (*n*=12,222). All images were normalized to the 0–255 intensity range and resized to 256×256 pixels. Labels were binarized into background (0) and airway (1) classes. To enhance generalization and address data imbalance, the following data augmentation techniques were applied during training: Flipping, both horizontally and vertically, was particularly useful in increasing dataset diversity and ensuring the model generalizes well to airways of different orientations. These preprocessing and augmentation strategies helped mitigate overfitting and improve model generalization.

### Experiments and Training

The proposed AirSeg module can be used as an encoder or a decoder on any UNet-like neural network base, making it flexible for plug-in and play. To test the inter-model performance of the AirSeg, we employed six UNet variants—UNet [35], UNet++ [36], ResUNet [37], VNet [38], SEUNet [39], and DenseUNet [40]. We integrated AirSeg as an encoder to these variants, and the decoder retained the corresponding variant’s decoder, providing the essence of the attentive encoder plus segmentation decoder. These integrations were compared with those of the same variants without AirSeg. Except for UNet, all other variants employed three to four Nested Skip Connections [41]. For the intra-model evaluation, we compared the proposed AirSegRes (AirSeg integrated with ResUNet architecture) with five other popular networks such as r2UNet [42], Dual attention network [43], Axial attention network [44], Attention UNet (AttnUNet) [45], and Pyramid attention network [46]. We set the number of heads in the IAM as 3 (the best results obtained with three heads). All models were trained and tested using the same protocol. We employed the Adam optimizer for training, utilizing a learning rate of 0.0001 and applying weight decay regularization with a coefficient of 0.00001 with a batch size of 32. For data augmentation, random rotation and flipping were performed to enhance the diversity of the training dataset. The models were trained for 200 epochs on a single NVIDIA P100 GPU with early stopping, combining the Dice loss and Intersection over Union loss as training losses [47]. On average, the proposed model took approximately 45 min to converge.

## Results

The weighted average percentage improvement improvements in DSC for all variants on the in vivo and in situ imaging datasets were 16.18% (*p*=0.0035) and 10.32% (*p*=0.0097), respectively, demonstrating statistically significant accuracy improvements when AirSeg was integrated with the variants on both datasets (see Table 1). Notably, the UNet++ variant showed limited improvement, suggesting that AirSeg performs better with residual nested skip connections than with dense nested skip connections. This finding indicates that further investigation is needed to comprehensively assess the performance of AirSeg in dense NSCs.

**Table 1:** Performance metrics of six conventional models with or without AirSeg on in vivo and in situ imaging datasets

Model	Dataset	DSC		IoU	
		With AirSeg	Without AirSeg	With AirSeg	Without AirSeg
UNet	In vivo	0.80 ± 0.24	0.55 ± 0.42	0.72 ± 0.25	0.49 ± 0.39
	In situ	0.70 ± 0.23	0.64 ± 0.22	0.57 ± 0.19	0.50 ± 0.18
UNet++	In vivo	0.80 ± 0.24	0.80 ± 0.24	0.72 ± 0.26	0.72 ± 0.26
	In situ	0.73 ± 0.23	0.68 ± 0.13	0.61 ± 0.20	0.53 ± 0.12
ResUNet	In vivo	0.81 ± 0.24	0.54 ± 0.41	0.73 ± 0.26	0.47 ± 0.38
	In situ	0.76 ± 0.15	0.62 ± 0.22	0.64 ± 0.15	0.48 ± 0.18
VNet	In vivo	0.80 ± 0.24	0.55 ± 0.43	0.72 ± 0.26	0.50 ± 0.40
	In situ	0.63 ± 0.21	0.62 ± 0.22	0.49 ± 0.17	0.47 ± 0.18
SEUNet	In vivo	0.80 ± 0.24	0.78 ± 0.23	0.71 ± 0.25	0.68 ± 0.24
	In situ	0.69 ± 0.10	0.64 ± 0.22	0.54 ± 0.10	0.50 ± 0.18
DenseUNet	In vivo	0.81 ± 0.24	0.60 ± 0.19	0.73 ± 0.26	0.45 ± 0.16
	In situ	0.66 ± 0.16	0.58 ± 0.15	0.51 ± 0.15	0.42 ± 0.13

Dice Similarity Coefficient (DSC) (mean±SD) and Intersection over Union (IoU) of conventional models with and without using proposed AirSeg for segmenting the airways on the unseen in vivo and in situ test imaging datasets

Similarly, Table 2 shows a weighted average accuracy improvement of 12.43% with AirSegRes compared to other models, achieving statistical significance (*p*=0.0004). The significantly low standard deviations observed for the proposed model and its variants further highlight the robustness and reliability of the model’s performance. These results underscore the model's efficacy in enhancing the encoder-generated features and capturing both long- and short-range semantic information.

**Table 2:** AirSegRes performance metrics compared to five other models

Model	DSC		IoU	
	In vivo	In situ	In vivo	In situ
r2UNet	0.43 ± 0.33	0.61 ± 0.21	0.33 ± 0.27	0.46 ± 0.17
Dual Attention	0.53 ± 0.36	0.58 ± 0.20	0.41 ± 0.28	0.43 ± 0.16
Axial Attention	0.47 ± 0.35	0.55 ± 0.25	0.37 ± 0.29	0.41 ± 0.20
AttnUNet	0.47 ± 0.36	0.64 ± 0.22	0.38 ± 0.30	0.50 ± 0.18
Pyramid Attention	0.63 ± 0.28	0.65 ± 0.22	0.52 ± 0.27	0.51 ± 0.18
AirSegRes	**0.81 ± 0.24**	**0.76 ± 0.15**	**0.73 ± 0.26**	**0.64 ± 0.15**

Dice Similarity Coefficient (DSC) (mean±SD) and Intersection over Union (IoU) of the proposed model, AirSegRes, in comparison to five conventional models for the airway segmentation on the unseen in vivo and in situ test imaging datasets

The observed difference in accuracy between the in vivo and in situ datasets could be attributed to the higher complexity of the in situ dataset. The in situ dataset contains a larger number of segmented regions (airways), introducing challenges such as class imbalance, heightened sensitivity to smaller and peripheral airways, and difficulties in distinguishing closely packed or overlapping regions. These challenges necessitate precise boundary delineation and make the smaller airways more difficult to detect due to their subtle appearance in CT images. Additionally, the variability in annotations further complicates segmentation.

**Fig. 3:**
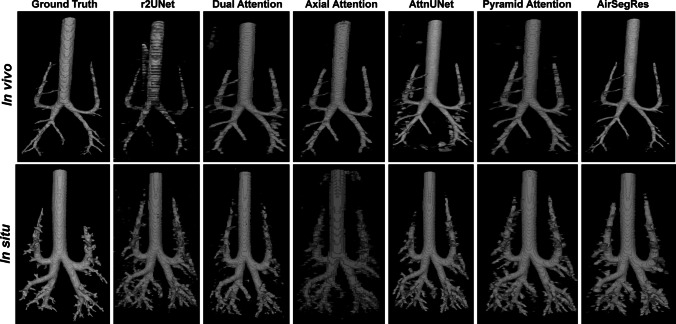
Representative airways segmentations. Representative segmentations as predicted by the proposed model (AirSegRes) in comparison to five other models such as r2UNet, Dual Attention, Axial Attention, AttnUNet, and Pyramid Attention along with the ground truth on in vivo and in situ imaging datasets

In contrast, the in vivo dataset, with fewer and more prominent airways, enables the model to leverage global context and simpler structural relationships, resulting in higher accuracy. Figure 3 provides a representative illustration of predictions from the models, while Table 3 presents an ablation study evaluating the impact of various attention mechanisms, embedding techniques, and fusion strategies on the Dice Similarity Coefficient (DSC) for the in vivo segmentation task. The results highlight how different architectural choices influence model performance and offer valuable insights into the contributions of individual components.

**Table 3:** Ablation study on in vivo dataset

Attention					Embedding			Fusion			DSC
Image	Position	Semantic	Spatial	Channel	Patch	CNN	New	Seq	Sum	Parallel	
✔							✔	✔			0.80
✔	✔	✔					✔	✔			0.80
✔			✔	✔			✔	✔			0.81
✔	✔	✔	✔	✔	✔			✔			0.79
✔	✔	✔	✔	✔		✔		✔			0.79
✔	✔	✔	✔	✔			✔	✔			0.81
✔	✔	✔	✔	✔			✔	✔			0.81
✔	✔	✔	✔	✔			✔		✔		0.80
✔	✔	✔	✔	✔			✔			✔	0.80

Dice Similarity Coefficient (DSC) on the in vivo test set on various ablated conditions like types of attentions, embedding, and fusion methods on AirSegRes

## Discussion

The AirSegRes demonstrates superior performance in airway segmentation by accurately delineating both primary airways and smaller, intricate branches, as demonstrated in Fig. 3. Unlike models such as r2UNet, Dual Attention, and Axial Attention, which often fail to capture fine details or produce incomplete segmentations, AirSegRes consistently preserves the structural integrity and closely matches the ground truth.

For the in vivo dataset, AirSegRes excels in capturing clear and continuous branch structures with minimal false negatives, outperforming models such as r2UNet, which frequently generates fragmented or overly smoothed predictions. The advanced attention mechanisms in AirSegRes enable dynamic focus on both global and local contextual features, ensuring precise segmentation even in challenging regions.

In the more complex in situ dataset, characterized by numerous small and peripheral airways, AirSegRes outperformed other models by accurately delineating these delicate structures. Other models often struggle with blurring or incomplete branching. Notably, AirSegRes's predictions sometimes appear thicker or include more branches than the ground truth, reflecting areas for potential enhancement. These discrepancies likely stem from the model's ability to amplify subtle airway structures, even in ambiguous regions, suggesting its potential for detecting details overlooked in manual annotations or constrained by CT imaging limitations. Future improvements could include advanced post-processing techniques, such as false-positive suppression or region-based filtering, to refine predictions and maintain segmentation accuracy for intricate airway structures.

Using Image Attention alone resulted in a DSC score of 0.8, indicating that focusing solely on high-level global features may not fully address the complexity of the segmentation task. Adding Position and Semantic Attention maintained the DSC score at 0.8, suggesting that these components alone do not significantly enhance performance. Incorporating Spatial and Channel Attention alongside these mechanisms increases the DSC to 0.81, demonstrating their effectiveness in capturing fine-grained and multi-dimensional features critical for accurate segmentation. The order of attention mechanisms applied did not produce a significant difference in performance.

Models utilizing the proposed Embedding Method achieve a DSC of 0.81, surpassing configurations with Patch or convolutional-based (CNN) embeddings. This indicates that the proposed embedding technique enhances feature representation by dynamically capturing spatial and semantic variations in the data. Further analyses were conducted to better understand the influence of the embedding method on the final output. While the DSC improvements in Table 3 may appear modest individually, they reflect the incremental contributions of each attention module and the embedding mechanism. Importantly, these components work synergistically, leading to a cumulative performance boost, especially in complex segmentation scenarios such as small airway branches. This combined improvement becomes more evident when comparing AirSeg-integrated models to baseline and other conventional architectures, where AirSeg consistently outperforms across multiple datasets. These gains, though incremental at the module level, translate into better sensitivity to fine structures and reduced manual correction, underscoring the clinical value of the proposed design.

We tested different values of $\epsilon \left({10}^{-3},{10}^{-4},{10}^{-5}\right)$ to understand its effect on numerical stability and feature sensitivity during variance normalization. A smaller $\epsilon \left({10}^{-5}\right)$ made the model highly sensitive to minor feature variations, which improved true positive detection of small and subtle regions such as peripheral airways. However, this increased sensitivity also led to more false positives, as noise and irrelevant features were amplified. Conversely, a larger $\epsilon \left({10}^{-3}\right)$ stabilized the variance calculation, reducing false positives by suppressing minor variations but occasionally causing under-segmentation by missing small or low-contrast structures. The intermediate value of $\epsilon \left({10}^{-4}\right)$ provided the best trade-off, offering consistent stability while maintaining sensitivity to critical features.

We experimented by varying the scaling factor in the formula, $\frac{{x}_{\mathrm{deviation}}}{k\cdot \left({\mathrm{variance}}+\upepsilon \right)}+0.5$, testing values of $k=2, 4, 8$. A smaller scaling factor ($k=2$) increased the sensitivity to variance, which enhanced true-positive rates for finer airway branches but resulted in over-segmentation, as noise and artifacts were amplified. On the other hand, a larger scaling factor ($k=8$) suppressed noise and improved false-positive suppression but struggled with detecting small features, leading to a reduction in true-positive rates. The default value $(k=4)$ provided a balanced outcome, improving both sensitivity and noise suppression, thereby achieving the highest overall segmentation accuracy.

The standard multiplicative scaling operation, ${x}_{\mathrm{scaled}}=x\cdot y$, was compared with an additive scaling approach, ${x}_{\mathrm{scaled}}=x+y.$ This allowed the model to emphasize high-confidence regions dynamically, leading to better true positive detection of relevant features while suppressing irrelevant areas. However, in some cases, it overamplified certain regions, slightly increasing false positives. In contrast, additive scaling provided more uniform adjustments, which avoided over-amplification but could not emphasize critical regions, reducing true positive rates sufficiently. A hybrid approach, combining multiplicative and additive scaling, was tested and showed potential for balancing these trade-offs, offering slightly improved segmentation in complex regions. Sequential fusion alone allowed us to consistently achieve a DSC of 0.81, indicating that processing features step-by-step may help preserve feature dependencies. Parallel fusion achieves a slightly lower DSC of 0.8, likely due to its inability to capture sequential dependencies effectively. AirSeg advances clinical workflows by precisely delineating airway anatomy, especially in hard-to-detect peripheral branches. This improved structural clarity supports earlier identification of respiratory conditions and enables more informed decision-making. Its adaptable design ensures easy integration into current imaging pipelines, enhancing both speed and consistency in diagnostic evaluations. Improved segmentation of peripheral airways has direct clinical implications. For example, enhanced detection of small airway branches can support early diagnosis of conditions such as bronchiolitis obliterans, a disease characterized by inflammation and obstruction of the smallest airways, which are often missed in routine analysis. Furthermore, precise airway maps are critical in planning and guiding bronchoscopic navigation, particularly for reaching peripheral lesions during biopsies or ablation procedures. By improving segmentation accuracy in these anatomically complex and clinically sensitive regions, we believe that AirSeg may reduce diagnostic delays and enhance procedural safety and precision.

Our study has several limitations. The relatively small sample size and lack of diverse datasets constrain the generalizability of the results. Incorporating larger, more diverse datasets with varied imaging conditions and demographics would provide a more comprehensive validation of the model performance. Additionally, while this study is centered on 2D networks, exploring 3D architectures could harness volumetric continuity to achieve improved segmentation outcomes, albeit at higher computational costs. Finally, the absence of comparisons with advanced architectures, such as transformer-based models, represents another limitation that could be addressed in future studies. While AirSeg has significantly improved airway segmentation, several directions can further enhance its performance and applicability. Future work will explore 3D architectures to capture volumetric airway structures better, reducing fragmentation and improving continuity in complex branching patterns. Integrating self-supervised learning could also leverage unlabeled data for pretraining, enhancing model generalization in data-scarce scenarios. Further research into domain adaptation techniques will help improve robustness across different imaging conditions and patient populations. Expanding AirSeg’s adaptability to real-time applications and refining its integration into clinical workflows will also be key areas of development.

## Conclusions

This study introduced AirSeg, a novel attention-based framework featuring a multifaceted attention mechanism coupled with a learnable embedding module to enhance airway segmentation in medical imaging. The proposed design effectively captures and integrates low- and high-level features, enabling precise localization of complex airway structures. By dynamically leveraging hierarchical feature representations, AirSeg excels in modeling both fine-grained details and broader contextual patterns, which is crucial for the accurate segmentation of small and intricate branches. The learnable embedding module further strengthens this process by enhancing feature interactions across network layers, contributing to improved segmentation performance. Looking ahead, AirSeg holds strong potential for integration into clinical workflows, including computer-aided diagnosis systems and automated airway analysis tools. Future work will focus on scaling the framework to 3D architectures, exploring self-supervised learning for data-efficient training, and validating performance across diverse clinical datasets to support broader real-world adoption.

## Data Availability

The data used in this study are proprietary and are not publicly available. Access to the dataset is restricted due to institutional regulations and confidentiality agreements. The use of data in this research has been reviewed and approved by the Institutional Animal Care and Use Committee (IACUC), ensuring compliance with ethical guidelines for the care and use of animals in research. Any requests for data access will be considered on a case-by-case basis and subject to institutional approval.

## Code Availability

The source code developed for this study is made publicly available in the following GitHub repository: https://github.com/chetana348/AirSeg-Learnable-Interconnected-Attention-Framework-for-Robust-Airway-Segmentation.
